# Biosorption of Strontium from Simulated Nuclear Wastewater by *Scenedesmus spinosus* under Culture Conditions: Adsorption and Bioaccumulation Processes and Models

**DOI:** 10.3390/ijerph110606099

**Published:** 2014-06-10

**Authors:** Mingxue Liu, Faqin Dong, Wu Kang, Shiyong Sun, Hongfu Wei, Wei Zhang, Xiaoqin Nie, Yuting Guo, Ting Huang, Yuanyuan Liu

**Affiliations:** 1 Key Laboratory of Solid Waste Treatment and Resource Recycle, Ministry of Education of China, Southwest University of Science and Technology, Mianyang 621010, China; E-Mails: dragonlmx@126.com (M.L.); zhangw@swust.edu.cn (W.Z.); 2 Life Science and Engineering College, Southwest University of Science and Technology, Mianyang 621010, China; E-Mails: weihongfu@swust.edu.cn (H.W.); ladyguoguo13@126.com (Y.G.); m15351254297@163.com (T.H.); 13778025425@163.com (Y.L.); 3 Institute of Nuclear Physics and Chemistry, China Academy of Engineering Physics, Mianyang 621900, China; E-Mail: kangwujy@126.com; 4 National Defense Key Discipline Laboratory of the Nuclear Waste and Environmental Safety of the Commission of Science, Technology and Industry for National Defense, Southwest University of Science and Technology, Mianyang 621010, Sichuan, China; E-Mail: xiaoqin_nie@163.com

**Keywords:** *Scenedesmus spinosus*, biosorption, adsorption, bioaccumulation, strontium, kinetics model

## Abstract

Algae biosorption is an ideal wastewater treatment method when coupled with algae growth and biosorption. The adsorption and bioaccumulation of strontium from simulated nuclear wastewater by *Scenedesmus spinosus* were investigated in this research. One hundred mL of cultured *S. spinosus* cells with a dry weight of 1.0 mg in simulated nuclear wastewater were used to analyze the effects on *S. spinosus* cell growth as well as the adsorption and bioaccumulation characters under conditions of 25 ± 1 °C with approximately 3,000 lux illumination. The results showed that *S. spinosus* had a highly selective biosorption capacity for strontium, with a maximum bioremoval ratio of 76%. The adsorbed strontium ion on cell walls was approximately 90% of the total adsorbed amount; the bioaccumulation in the cytoplasm varied by approximately10%. The adsorption quantity could be described with an equilibrium isotherm. The pseudo-second-order kinetic model suggested that adsorption was the rate-limiting step of the biosorption process. A new bioaccumulation model with three parameters was proposed and could give a good fit with the experiment data. The results suggested that *S. spinosus* may be a potential biosorbent for the treatment of nuclear wastewater in culture conditions.

## 1. Introduction

The biosorption of heavy metals by living or growing biomasses involves two processes: an initial rapid, passive adsorption followed by a much slower active bioaccumulation process. The former is a metabolism-independent process, while the latter is metabolism-dependent [1,2]. The majority of studies on the bioaccumulation of heavy metal ions by microorganisms only consider the final concentration of metal ions, and only a few studies provide quantitative kinetic descriptions of the process and investigate its mechanisms [3]. Thus, the investigation of adsorption and bioaccumulation processes and their mechanisms can facilitate application of these techniques in wastewater treatment or other associated fields. 

Krejci *et al.* [4] have found that a common freshwater alga (*Closterium moniliferum*) showed the notable ability to bioremove strontium from water and proposed a “sulfate trap” model by preferential precipitation of (Ba, Sr)SO_4_ due to its lower solubility compared to SrSO_4_ and CaSO_4_. It is believed that this discovery can help scientists design suitable methods to bioremove radioactive strontium from existing nuclear wastewater streams as many algae have immense metal ion biosorption capabilities [2]. The metal biosorption capacity of an algal biomass is comparable, or sometimes higher, than that of chemical sorbents [2,5]. In addition to the higher biosorbing capacity, algae are autotrophs, which can grow in wastewater with little or no nutritional supplements [2]. The coupling of the algal growth and biosorption of harmful metal ions from wastewater under culture conditions make algal biosorption an ideal metal ion scavenging method [6,7]. *Scenedesmus spinosus* is a common type green alga that typically consists of 4–8 cells aligned in a flat plate with single short spine arising at each pole of terminal cells and one spine at the middle of terminal cell [8]. *S. spinosus* is abundant in many natural water environments and shows good tolerance to heavy metal pollution [9,10,11]. *S. quadricauda* is commonly used for a variety of studies in different fields of science [5], but there are few publications regarding *S. spinosus*. 

There are some published studies that investigated the capacity of *Scenedesmus* to biosorb heavy metal ions from solutions. *S. quadricauda* showed a highly efficient sequestration capacity of Ni^+^ and Cu^2+^ [2,12]. Terry and Stone [10] have shown that while both living and nonliving *S. abundans* removed cadmium and copper from water, living algae significantly outperformed nonliving algae. Omar [13] has found that the maximum specific adsorptive capacity of zinc ions obtained from the Langmuir adsorption isotherms was higher for *S. obliquus* (6.67) and lower for *S. quadricauda* (5.03) and that *S. obliquus* was more tolerant of zinc phytotoxicity than *S. quadricauda*. A Wuhan isolate of *Scenedesmus* could tolerate a mixture of 30 mg∙L^−1^ Ni^2+^ and 30 mg∙L^−1^ Zn^2+^ in wastewater and could remove most of the Ni^2+^ and Zn^2+^ from the simulated wastewater in 5 min [14]. *S. quadricauda* had a high biosorption capacity for Ni^2+^, Cu^2+^ and Cu^+^, while the biosorption capacity was lowest for Mo^6+^ of the six metals (Cu^2+^, Cu^+^, Mo^6+^, Mn^2+^, V^5+^, Ni^2+^), both individually and when in combined solutions of these metals [15]. The immobilized *S. quadricauda* showed a good adsorption capacity for Cu^2+^, Zn^2+^ and Ni^2+^ ions and was particularly selective for Cu^2+^ ions [5,16]. 

Biosorption with microorganisms under culture conditions may facilitate the development of continuous industrial treatment of contaminated wastewater [17]; however, as described above, there are few publications concerned with the treatment of liquid wastes with low or intermediate levels of radioactivity with *S. spinosus* under culture conditions. 

The present research has investigated the bioremoval efficiency of strontium ions from the simulated nuclear wastewater by *S. spinosus* under culture conditions and analyzed the adsorption and bioaccumulation characteristics, processes, kinetics and models.

## 2. Experimental Section

### 2.1. Scenedesmus spinosus and Culture Method

The *Scenedesmus spinosus* was isolated from the wastewater samples which were obtained from the Tianfu Changcheng pool, Chengdu, China. The culture medium was a modified Chu No. 10 nutrient solution: Ca(NO_3_)_2_ 0.04 g; K_2_HPO_4_ 0.01 g; MgSO_4_·7H_2_O 0.025 g; Na_2_CO_3_, 0.02 g; NaSiO_3_ 0.025 g; Soil extract 20 mL; F/2Vitamin stock solution, 1 mL; water, 1,000 mL; pH 6.5–7.0. The culture was incubated statically at 25 ± 1 °C. Incubation was performed under a continuous light intensity of approximately 3,000 lux, which was produced by two 40-W white fluorescent lamps placed above the Erlenmeyer flasks. During the culturing period, the Erlenmeyer flasks containing the cultures were shaken twice a day, the culture medium was replaced every 3 days, and the pH was allowed to drift freely. Culturing was performed over a period of approximately 7 days, and the cultured *S. spinosus* was then prepared for biosorption.

### 2.2. Reagents

All chemical reagents used were of analytical grade. The strontium stock solution (500 mg∙L^−1^) was prepared by dissolving 1.2575 g of strontium nitrate (Sr(NO_3_)_2_) in 1,000 mL of distilled deionized water. A simple solution containing only strontium ions was prepared by diluting the stock solution to a preset concentration.

The components of the simulated low- and intermediate-level radioactive liquid waste (simplified as simulated wastewater) are listed in Table 1 [18]. In this research, the strontium concentration was reduced below the reference data because when the strontium concentration is larger than 200 mg∙L^−1^, other constituents in the medium, such as sulfate, carbonate and phosphate ions, may react with strontium ions to form sediments [19]. The strontium ion concentration was varied from 0 to 100 mg∙L^−1^ to analyze the effects of coexisting ions on the biosorption of strontium. 

**Table 1:** The simulated low and intermediate level radioactive liquid waste components.

Elements	Nitrates	Contents/g∙L^−1^
Na	NaNO_3_	31.560
Al	Al(NO_3_)_3_	13.670
Fe	Fe(NO_3_)_3_	7.460
Cr	Cr(NO_3_)_2_	0.960
Ni	Ni(NO_3_)_2_	2.670
K	KNO_3_	0.620
Ba	Ba(NO_3_)_2_	0.014
Sr	Sr(NO_3_)_2_	0–0.1
Cs	CsNO_3_	0.175

### 2.3. Biosorption of Strontium Ions by S. spinosus under Batch Culture Conditions

The simple solutions or simulated wastewater solutions (20 mL) were mixed with suspensions of growing *S. spinosus* (40 mL, cell dried weight: 1.0 mg∙L^−1^) and the culture medium (40 mL) prepared as described above. Then, the mixture continued to culture. After a preset biosorption time (11 time gradients from 1 to 144 h for simple solutions and six time gradients from 24 to 144 h for simulated wastewater solutions), the cell suspensions were centrifuged (22 °C, 15 min, 4,000 rpm). The supernatants were collected for analysis of the residual metal ion concentrations, and the sediment was collected for analysis of the adsorption and bioaccumulation of strontium ions by *S. spinosus* cells. 

### 2.4. The Adsorption and Bioaccumulation of Strontium on S. spinosus Cells Analysis with Wet Ashing Method

A brief description of the wet ashing method follows [20]. First, the *S. spinosus* cell sediments collected after the biosorption phase were washed with distilled, de-ionized water to remove all of the unbound strontium ions from the *S. spinosus* cells. After washing, each sediment sample was suspended in 2 mL of 0.01 mol∙L^−1^ EDTA and agitated for 5–10 min, and then the mixtures were centrifuged for 20 min at 10,000 rpm. The supernatants were then collected. The centrifugation and supernatant collection process was repeated twice, and the three supernatant samples collected were then combined to measure the strontium ion concentration adsorbed by the *S. spinosus* cell walls. The cell sediments collected after washing with EDTA were digested with 2 mL of nitrification solutions (HClO:HNO_3_ = 1:4) for 48 h. After digestion, the mixtures were centrifuged for 20 min at 10,000 rpm; the supernatants were then collected to measure the concentration of strontium ions intracellularly bioaccumulated by the *S. spinosus* cells. 

### 2.5. Metal Ions Concentration Measurement and Data Analysis

Timed sample supernatants of 10 mL were used to assay residual metal ion concentrations with an atomic absorption spectrophotometer (PE AA700, Shelton, CT, USA) at the Analytical and Testing Center, Southwest University of Science and Technology. The bioremoval ratio (*R*) was calculated using the following formula:

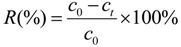
(1)


The biosorption quantity of strontium (*q*) was calculated with the following equation:

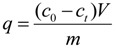
(2)
where *q* (mg∙g^−1^) is the amount of strontium adsorbed per gram of dry weight, *c*_0_ (mg∙L^−1^) is the initial concentration of strontium, *c*_t_ (mg∙L^−1^) is the concentration of the strontium in solution at the preset culture time, *V* (L) is the volume of solution, and *m* (g) is the dry mass of *S. spinosus*.

The quantity of strontium ions adsorbed in the *S. spinosus* cell walls was calculated using the following equation:

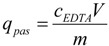
(3)
where *q*_pas_ (mg∙g^−1^) is the adsorption quantity of strontium ion in the *S. spinosus* cell walls, *c_EDTA_* (mg∙L^−1^) is the strontium concentration in the EDTA washing solution, *V* (L) is the volume of the EDTA washing solution, and *m* (g) is the dry mass of *S. spinosus*.

The quantity of strontium ions bioaccumulated in the *S. spinosus* cytoplasm was calculated as follows:

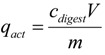
(4)
where *q*_act_ (mg∙g^−1^) is the quantity of strontium ions bioaccumulated in the *S. spinosus* cytoplasm, *c_digest_* (mg∙L^−1^) is the strontium concentration in the digest solution, *V* (L) is the volume of the digest solution, and *m* (g) is the dry mass of *S. spinosus*.

When performing calculations for the bioaccumulation model, *c*_t_, *q*_pas_ and *q*_act_ were written as *c*_Me_, *c*_pas_ and *c*_act_, which represent the free strontium ions in solution, the strontium ion concentrations on the *S. spinosus* cell walls and the strontium ion concentrations in the *S. spinosus* cytoplasm, respectively. 

### 2.6. Modeling

The parameters of the nonlinear isotherm models and bioaccumulation model were determined by linear programming; the arithmetic used is as follows:

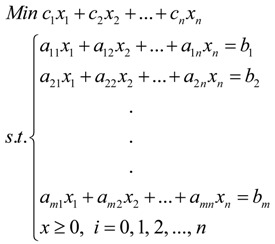
(5)


## 3. Results and Discussion

### 3.1. The Effects of Strontium Ions in Simulated Wastewater on S. spinosus Cell Growth

**Figure 1:**
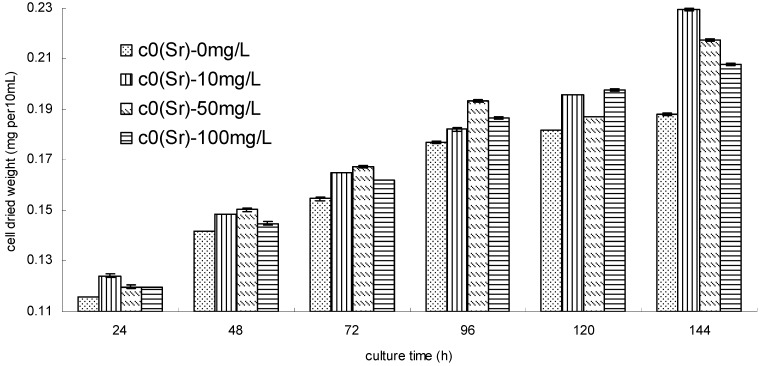
The effect of simulated wastewater on *S. spinosus* growth during the biosorption phase (*S. spinosus* cell dried weight per 10 mL of culture medium).

Observation under a microscope showed that the *S. spinosus* cells that were separated from the wastewater grew well in the simulated nuclear wastewater. The results in Figure 1 show that the low concentration of strontium ions in the simulated wastewater could stimulate the growth of *S. spinosus* cells. The cell dried weight approximately doubled in the 144-h culture stage. A similar result was obtained for *Dicratetia inornata* and *Platymonas subcordiformis*, which can tolerate strontium ion concentrations greater than 1.44 mmol∙L^−1^ [21,22]. The *D. inornata* growth was severely inhibited when the initial strontium concentration reached 5.76 mmol·L^−1^ (approximately 500 mg∙L^−1^) [21]. The *S. spinosus* growth stimulation by the simulated wastewater may be due to the high concentrations of ferric, sodium and other ions. Liu *et al.* [23] have shown that a ferric ion concentration of 3,000 nmol∙L^−1^ could also enhance the growth of *S. quadricauda* cells.

### 3.2. The Bioremoval Ratio of Strontium Ions from Simulated Wastewater by S. spinosus under Batch Culture Conditions

**Figure 2:**
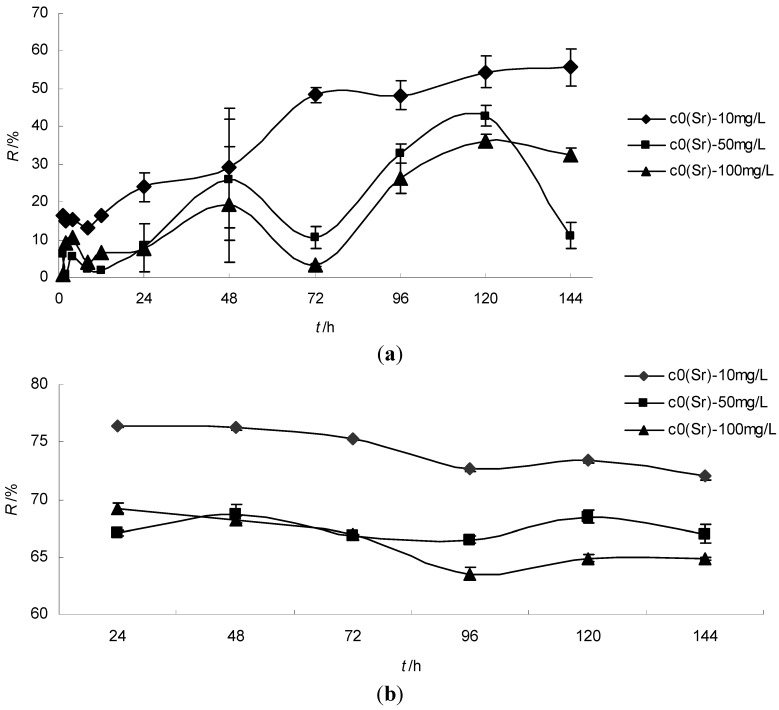
(**a**) The bioremoval ratio of strontium ions from a simple solution containing single strontiumions by *S. spinosus* under batch culture conditions; (**b**) The bioremoval ratio of strontium ions from simulated wastewater by *S. spinosus* under batch culture conditions.

To investigate the biosorption of strontium ions from the simulated nuclear wastewater, biosorption of strontium ions from a simple solution containing a single strontium ion was first carried out (Figure 2a). The results showed that the bioremoval ratio of strontium ions varied with the culture time. The bioremoval ratio of strontium ions increased with culture time when the initial strontium ion concentration was 10 mg∙L^−1^; a maximum removal ratio of approximately 60% was obtained. The strontium ion removal behavior was similar for initial strontium ion concentrations of 50 mg∙L^−1^ and 100 mg∙L^−1^. The results suggested that the *S. spinosus* had a low efficiency for bioremoval of strontium ions from a simple solution. 

Because the bioremoval ratio was very low during the first 24 h for strontium ion bioremoval from the simple solution, a process for the biosorption of strontium ions from simulated wastewater was designed from 24 to 144 h. 

The results showed that the bioremoval of strontium ions from simulated wastewater was different compared to that from a simple solution. Figure 2b shows that the removal of strontium ions was a quick process; the biosorption reached equilibrium in 24 h, and the removal ratio was above 60%, with a maximum removal ratio of approximately 76%. For an initial concentration of 10 mg∙L^−1^, the removal ratio increased with the culture time in simple solution conditions, while it decreased in simulated wastewater. For initial concentrations of 50 and 100 mg∙L^−1^, a periodic oscillation of the removal ratio, with a period of approximately 72 h, was observed. A similar trend was observed for the biosorption of Cd^2+^ by *Chlorella pyrenoidosa* [24]. 

### 3.3. The Co-Existing Cations Effects on Biosorption of Strontium Ions in Simulated Wastewater

Factors that affect biosorption include pH, ion mixing, time, temperature, pretreatment and biomass quantity. Co-existing metal ions mainly affect the biosorption of strontium ions from wastewater [25]. In this research, the co-existing cations were treated as a whole; the strontium ion concentration was varied to analyze the co-existing cations’ effect on strontium ion adsorption and bioaccumulation.

**Figure 3:**
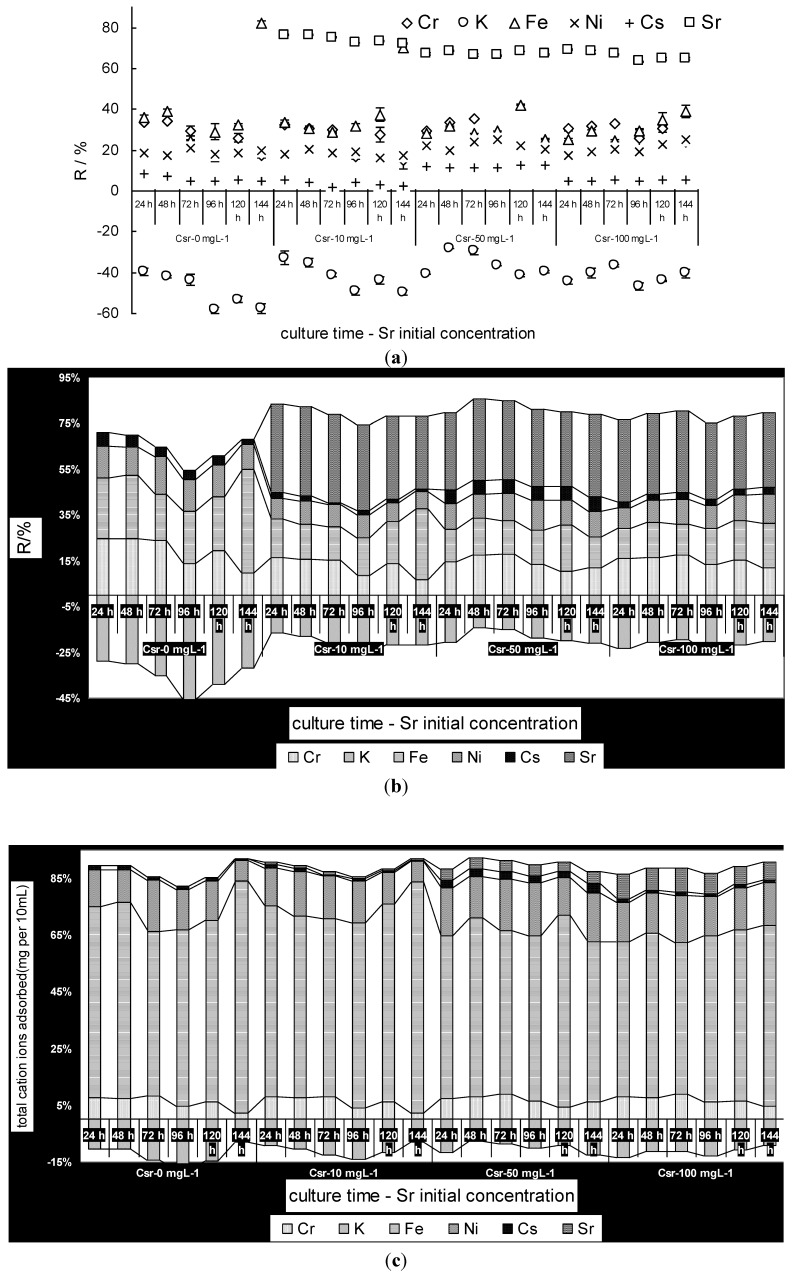
(**a**) The packing arrangement figure of the co-existing cation bioremoval ratio (*R*); (**b**) The packing arrangement figure of adsorbed cation quantity per 10 mL (*q*); (**c**) The total adsorbed cation quantity per 10 mL (*q*).

The various co-existing cations investigated in simulated wastewater showed different effects on strontium ion biosorption in this study. The *S. spinosus* cells displayed a great bioremoval ratio for strontium, ferric and chromium ions; a negative bioremoval ratio for potassium ions; and a small bioremoval ratio for nickel and cesium ions (Figure 3a). During the first 72-h biosorption course, the strontium ions caused the bioremoval ratio of the other cations to decrease. Additionally, the bioremoval ratios varied with the initial strontium ion concentration. During the next 72-h biosorption course, the strontium ions increased the bioremoval ratio of the other cations. A periodic oscillation of the bioremoval ratios was seen for other cations, similar to that of the strontium ions. The total ion quantity adsorbed by *S. spinosus* when strontium ions were added to the simulated wastewater was more stable than the quantity with no strontium ions in the simulated wastewater during the 144-h bisorption course. The total ion quantity adsorbed increased greatly in the 144-h biosorption course due to the large increase in the biosorption of ferric ions. The packing arrangement figure (Figure 3b,c) of adsorbed ions quantity per 10 mL showed that the ferric ions were the largest cations, followed by nickel and chromium ions. *S. spinosus* cells adsorbed few cesium ions. The strontium ion concentration was the lowest in the simulated wastewater (below 0.1 g∙L^−1^). However, the *S. spinosus* cells had a high bioremoval ratio for strontium ions, and a relatively high adsorption quantity. These results suggested that the *S. spinosus* cells showed a strong selective biosorption capacity for strontium ions. The other cations’ bisorption was dependent on the ion concentration. The negative removal ratio of potassium ions suggests that biosorption of other metal ions caused an ion-exchange with potassium ions. The small biosorption quantity for cesium ions may be due to the small bisorption capacity for monovalent cations. The results showed that the total biosorbed ion quantity increased with the initial strontium ion concentration, and the quantity in the different culture stages was almost at the same level. This suggested that the biosorption capacity of *S. spinosus* of metal ions was dependent on the ions’ concentrations in the solution. 

The grey relational grade analysis showed that the order for different co-existing metal ion effects on strontium ion biosorption was Fe^3+^ > Cr^3+^ > Ni^2+^ > K^+^ > Cs^+^.

The biosorption of metal ions by algae is a larger concern for single, heavy metal ions such as Cd^2+^, Ni^2+^, Cr^3+^, Cu^2+^ [24,26,27]. These studies also agree with the present research results that the amount of metal ions absorbed by the alga increased with higher initial metal ion concentrations [24,26,27]. The sorption capacity of the chlorella for Cd^2+^ was much higher than that for Cu^2+^ and Cd^2+^ [28]. Increasing the Cu^2+^ concentration could increase the biosorption of Cr^4+^ by *Rhizopus nigricans* [29]. However, prior research has shown the poor performance of metal ions in simulated wastewater. In the present research, the performance of multi-metal ions in simulated wastewater was analyzed. The results showed that the *S. spinosus* displayed a great bioremoval ratio for strontium ions compared to other co-existing cations’ bioremoval ratio. The reason may be that metal ions of Fe^3+^ in the simulated wastewater could promote the *S. spinosus* growth [23], thereby increasing the strontium scavenging ratio compared to simple solutions. The toxicity of Ni^2+^, Cr^2+^ and Cs^+^ metal ions [26] not only showed a low inhibiting effect on the growth and morphology of *S. quadricauda* but also caused a low scavenging ratio by *S. quadricauda* cells. That is, that single poisonous metal ion was no more toxic when combined in the same system. This may explain why many algae can naturally grow well in polluted waters. This also proved the opinion that actinide toxicity is primarily chemical (not radiological) and that radiation resistance does not ensure radionuclide tolerance [30]. Because the radioactivity of low- and intermediate-level radioactive liquid waste is low, algae can endure the chemical toxicity and grow well in these media; this fact facilitates the application of algae for radioactive nuclides wastewater treatment. 

### 3.4. The Adsorption and Bioaccumulation of Strontium Ions by S. spinosus Cells

**Figure 4:**
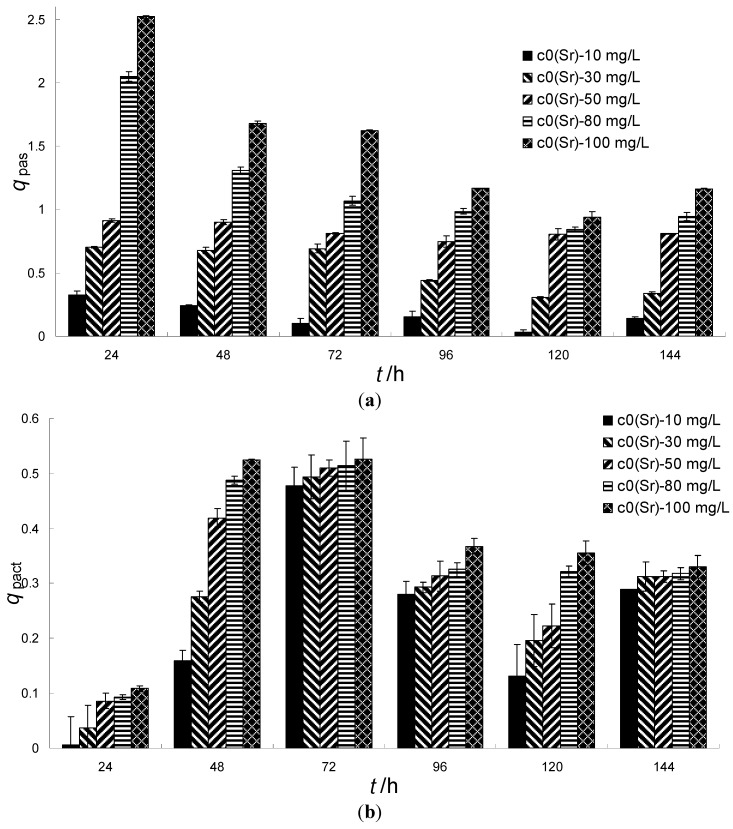
(**a**) The adsorption of strontium ions by *S. spinosus* cells from simulated wastewater (*q*_pas_-*t*); (**b**) The bioaccumulation of strontium ions by *S. spinosus* cells from simulated wastewater (*q*_act_-*t*).

The cell walls of algae have the capacity to bind metal ions in negatively charged sites [31,32]. However, the algae also have the ability to accumulate the metal ions through metabolism under culture conditions. Figure 4a showed that the adsorption of strontium ions by *S. spinosus* was a very rapid process when the *S. spinosus* was cultured in simulated wastewater. The *q*_pas_ reached its peak in the first 24 h, which suggests that the adsorption process is a physical reaction that occurs early in the adsorption course and the cell walls were the first sites for adsorbing. When the biosorption process continued, *q*_pas_ decreased with culture time. The *q*_pas_ value also increased with the initial strontium ion concentration at different culture stages. The adsorption process can be divided into two stages: during the first 72-h stage, the *q*_pas_ was high; during the next 72-h stage, the *q*_pas_ value was low. This change may be explained as follows: (1) The adsorbed strontium ions were transported into the cytoplasm through metabolism; (2) The cell weight increased. For example, the cell weight increased from 0.09 g per 10 mL at 24 h to 0.24 g per 10 mL at 144 h; (3) The cell may have acquired a resistance to strontium ions that led to desorption of adsorbed strontium ions. 

The bioaccumulation *q*_act_ was very small during the first 24 h and increased during the next 48 h (Figure 4b). In the following 72 h, *q*_act_ decreased noticeably. In general, *q*_act_ increased with the initial strontium ion concentration, but with longer residence times, this correlation diminished. For example, *q*_act_ reached nearly the same value after 72 h and 144 h. This suggested that the bioaccumulation of metal ions did not depend on the initial metal ion concentration, and the bioaccumulation *q*_act_ may mainly depend on the metabolism of the cell and the cell’s tolerance of the metal ions. In the first 72 h, the main reaction was bioaccumulation through metabolism, so *q*_act_ increased rapidly. The decrease in *q*_act_ may be due to the decrease in *q*_pas_ described above. Thus, these results suggested that the biosorption of metal ions may be a time-dependent process under culture conditions, and an optimal biosorption time existed that varied with the biosorbent and metal ion types, which is different from biosorption by the traditional method. The similar results were obtained from biosorption of cadmium by *C. pyrennoidosa*. The uptake of cadmium by *C. pyrennoidosa* occurred in at least two phases: fast (8 min) and slow (24 h). The fast phase consisted of ion-exchange uptake onto the cell wall, while the slow phase involved transport across the cell membrane [24].

The different initial strontium ion concentrations could yield nearly the same level of *q*_act_, suggesting that the cell had a saturated bioaccumulation quantity that did not depend on *q*_pas_. The decrease in *q*_act_ may be due to the cell initially developing accommodation sites for the ions and then opening an ion channel to transport the strontium ions out of the cytoplasm.

The data also showed that the adsorbed strontium ions on the cell wall occupied over 90% of the available ions early in the biosorption process and then decreased to approximately 70% later in the biosorption stage. Latha *et al.* [33] showed that the *Neurospora crassa* surface accommodates approximately 90% of cobalt ions. *Candida utilis* cell walls accumulated 50% of the copper ions and it was suggested that the cell wall was the main site for heavy metal accumulation [34]. The La^3+^, Eu^3+^, Yb^3+^ lanthanide ions that accumulated on *Pseudomonas aeruginosa* cell walls accounted for approximately 90% of the total ion uptake [35]. However, it was interesting to find that the intracellular strontium ratio for a low initial strontium concentration was larger than that for a high initial strontium concentration. The reason for this result may be that the low concentration strontium is less toxic to *S. spinosus* cells, giving the *S. spinosus* cells a larger bioaccumulation capacity. 

### 3.5. Equilibrium Isotherm

The capacity of a biosorbent can be described by certain constants of the equilibrium sorption isotherm, the values of which express the surface properties and affinity of the biosorbent [36]. To analyze the validity of the adsorption data, the most common adsorption isotherm models (Langmuir, Freundlich, *etc.*) were applied, as discussed below. The common biosorption equilibrium isotherm was modeled with *q*_e_ data calculated according to Equation (2). In this study, we have assayed the cell wall adsorption quantity *q*_pas_. Thus, we fit the equilibrium isotherm with *q*_pas_. 

The Langmuir equation and linear expression were respectively describled as follows [37]:

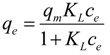
(6)

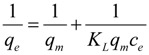
(7)
where *q*_e_ is the amount adsorbed (mg∙g^−1^), *c*_e_ is the equilibrium concentration of the adsorbate (mg∙L^−1^), *q*_m_ is the Langmuir constants related to the maximum monolayer adsorption capacity (mg∙g^−1^), and *K*_L_ is the constant related to the free energy or net enthalpy of adsorption. 

The Freundlich isotherm equation and logarithmic form were respectively describled as follows [38]:

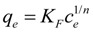
(8)

ln *q_e_* = ln *K_F_* + (1 / *n*)ln *c_e_* (9)

where *K*_F_ (L∙g^−1^) and *n* indicate the affinity of the adsorbate to the biomass. Therefore, the values of *K*_F_ and 1/*n* were calculated from the plots of ln *q*_e_ *versus* ln *c*_e_. 

The three-parameter isotherm model Koble-Corrigan equation was describled as follows [39]:

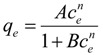
(10)
where *A* (L·g^−1^), *B* and n were the Koble-Corrigan isotherm parameters. 

Besides the *r*^2^, Chi-square test was adopted to assess the suitability of selected isotherm model. The Chi-square test equation was described as follows [40]:

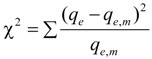
(11)
where *q*_e,m_ (mg·g^−^^1^) is the calculated adsorbed amount based on the model parameters.

The Langmuir biosorption isotherm was more suitable for the estimation of the monomolecular maximum adsorption capacity. The monomolecular adsorption capacity *q*_m_ (Table 2) showed that the *S. spinosus* cell walls had nearly the same adsorption capacity in different culture stages. The dimensionless equilibrium parameter (*R*_L_) was used to estimate the affinity between the sorbate and sorbent [41]. The RL expression is:

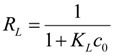
(12)
where *K*_L_ is the Langmuir constant and *c*_0_ is the initial strontium ion concentration. The different value of *R*_L_ indicated the different type of Langmuir isotherm: irreversible (*R*_L_ = 0), linear (*R*_L_ = 1), unfavorable (*R*_L_ > 1), or favorable (0 < *R*_L_ < 1) [41]. The *R*_L_ value was from 0.28952 for *c*_0_ 100 mg∙L^−1^ at 24 h to 0.85049 for *c*_0_ 10∙mg·L^−1^ at 48 h. These results indicated that the adsorption of the strontium ion onto *S. spinosus* cells is favorable [41]. The parameters in Table 2 regressed with linear or nonlinear method suggested that the linear isotherm regression and nonlinear isotherm regression both completely fitted the experimental data based on *r*^2^ and *χ*^2^.

**Table 2:** The equilibrium isotherm model parameters.

Model	Time (h)	Linear Isotherm Regression				Nonlinear Isotherm Regression				
		Parameters		*r*^2^	*χ*^2^	Parameters			*r*^2^	*χ*^2^
		*q*_m_	*K*_L_			*q*_m_	*K*_L_			
Langmuir	24	2.35627	0.06618	0.9531	0.8864	7.65490	0.01374		0.9262	0.1902
	48	2.34082	0.04904	0.9966	0.0594	4.52553	0.01758		0.9780	0.0498
	96	2.62950	0.02242	0.9998	0.0019	2.44126	0.02518		0.9985	0.0018
	144	2.50188	0.02127	0.9868	0.0597	2.89086	0.01908		0.9668	0.0413
Freundlich		*n*	*K*_F_			*n*	*K*_F_			
	24	1.29702	0.43837	0.9311	7.6716	0.85574	0.04543		0.9548	0.4544
	48	1.39179	0.41849	0.9924	6.8841	1.35220	0.12624		0.9885	0.0160
	96	1.25565	0.31886	0.9922	9.4048	1.43487	0.09741		0.9910	0.0156
	144	1.16918	0.29145	0.9748	10.8071	1.32003	0.07969		0.9581	0.0556
Koble-Corrigan						*A*	*B*	*n*		
	24					0.03265	−0.89004	0.02991	0.9721	0.0744
	48					0.16437	−0.10717	0.45695	0.9928	0.0171
	96					0.05230	0.02496	1.08766	0.9988	0.0015
	144					0.02130	0.01346	1.47458	0.9719	0.0568

The Freundlich biosorption isotherm was based on the biosorption of a heterogeneous surface. The values of *K*_F_ were found to be decreasing with culture time. The nonlinearity degree between metal ion concentration and adsorption can be determined by n value: linear (*n* = 1), poor adsorption capacity (*n* < 1), good adsorption capacity (*n* > 1) [41]. The *n* value showed in Table 2 was 1.16918–1.39179. The linear isotherm regression values of *n* > 1 indicated positive binding and a heterogeneous nature of adsorption of the *S. spinosus* cell walls [41]. The *r*^2^ results (Table 2) showed that the linear isotherm regression and linear isotherm regression both fitted the experimental data. But the *χ*^2^ results suggested that the nonlinear isotherm regression was more suitable for accurately describing the Freundlich isotherm relationship than the linear isotherm regression.

Compared to two-parameter isotherm models, the three-parameter isotherm model Koble-Corrigan was the best fitted isotherm model based on *r*^2^ and *χ*^2^ parameters. These results suggested that the strontium ion biosorption onto *S. spinosus* cells was a complex process following a combined isotherm models [42].

The cell wall is the main site for biosorption of metal ions, but the cell may transport some metal ions into the cell’s interior under culture conditions by metabolism. Thus, the equilibrium isotherm model should be described using the higher resolution data of the cell wall-adsorbed ion quantity instead of the total biosorption quantity [1]. For example, Ergene *et al.* [43] elucidated the equilibrium isotherm model of immobilized active and inactive *S. quadricauda* for dye removal using the *q*_e_, which was calculated based on the changes in the initial and equilibrium ion concentrations. The *q*_pas_ data were used to elucidate the equilibrium isotherm model in the present research. The results suggested that the model could be used to evaluate the metal ion adsorption on the cell surfaces. The results also indicated that the adsorption of the metal ions by *S. spinosus* cell walls followed the isotherm laws. It was showed that the nonlinear isotherm regression method is a better way to obtain the isotherm parameters.

### 3.6. Kinetic Models for Biosorption

To determine the metal ion biosorption kinetics, the Ritchie pseudo-second-order kinetic model was used; the equation is shown below [44,45,46]:

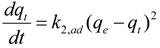
(13)


Integrating and rearranging with the initial condition *q*_t_ = 0 at *t* = 0, the following equation is obtained:

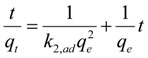
(14)
where dq_t_/dt is the initial biosorption rate (mg∙g^−1^∙min^−1^) and is defined as *t*→0 by *h* = *k*_2,ad_*q*_e_^2^, and *k*_2,ad_ is the pseudo-second-order biosorption rate constant (g∙mg^−1^∙min^−1^). The value *q*_e_ is determined from the slope of *t*/*q*_t_ *versus t* and *h* is determined from the intercept [44].

The Lagergren pseudo-first-order and Ritchie pseudo-second-order kinetic models were used for estimation of the biosorption kinetics [47,48]. The data for the present research did not fit the Lagergren pseudo-first-order model, so the data are not included in Table 3. To analyze the biosorption kinetics, *q*_e_ based on the scavenging ratio data, *q*_pas_ based on the adsorption data and *q*_pas+act_ based on the adsorption adding bioaccumulation data were used to fit the model. The rate parameters for the biosorption of the strontium ions by the *S. spinosus* cells are presented in Table 3. Analysis of *r*^2^ showed that the data based on the scavenging ratio was more suitable for the pseudo-second-order model than the data based on adsorption and adsorption with bioaccumulation. This result suggested that the model was more suitable for describing the total ion changes in the adsorption system. This result indicated that adsorption may be the rate-limiting step of the biosorption process [43].

**Table 3:** The kinetic model parameters.

Calculation Model	The Kinetics Model Parameters				
Based on scavenging ratio(*R*)	*c*_0_(sr)	*q*_e_	*k*_2_	*h*	*r*^2^
	10 mg∙L^−1^	0.29345	−0.17085	−0.01471	0.9740
	50 mg∙L^−1^	1.45264	−0.04289	−0.09050	0.9831
	100 mg∙L^−1^	2.80110	−0.02057	−0.16139	0.9932
Based on *q*_pas_+*q*_act_	*c*_0_(sr)	*q*_e_	*k*_2_	*h*	*r*^2^
	10 mg∙L^−1^	0.25839	−0.26045	−0.01739	0.4950
	50 mg∙L^−1^	1.04998	−0.18523	−0.20420	0.9759
	100 mg∙L^−1^	1.23870	−0.04302	−0.06600	0.9620
Based on *q*_pas_	*c*_0_(sr)	*q*_e_	*k*_2_	*h*	*r*^2^
	10 mg∙L^−1^	0.05534	−0.69997	−0.00214	0.3631
	50 mg∙L^−1^	0.77286	−0.35743	−0.21350	0.9937
	100 mg∙L^−1^	0.92618	−0.05466	−0.04688	0.9465

### 3.7. Models for Bioaccumulation

The pseudo-second-order model was well suited for description of the total ion concentration change kinetics, and the equilibrium models were suitable for the description of the cell wall adsorption quantity. There are only a few models describing bioaccumulation in the literature [3]. A two-step bioaccumulation process was proposed that afforded a quick binding of metal ions to the cell walls, followed by a slower transport process through the cell membrane [3]. 

Based on the following physical model, Chojnacka and Wojciechowski [3] proposed a mathematical kinetics model:

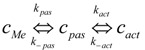
(15)


On the basis of microscopic observations, it was observed that during microbial growth, the ratio of cellular mass to their surface area is constant. The equation of mass balance and kinetic equations describing concentrations of all forms of strontium ions were proposed:

*c_Me_* (*t*) + *c_pas_* (*t*) + *c_act_* (*t*) = *c_Me_* (*t* = 0) (16)

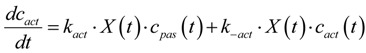
(17)


However, when this model was used to analyze the experimental data, a good mathematical model could not be obtained. From Equation (17), the following bioaccumulation kinetics model was proposed based on the biosorption and bioaccumulation mechanisms in the present study:
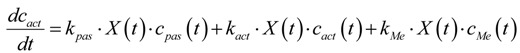
(18)


**Figure 5:**
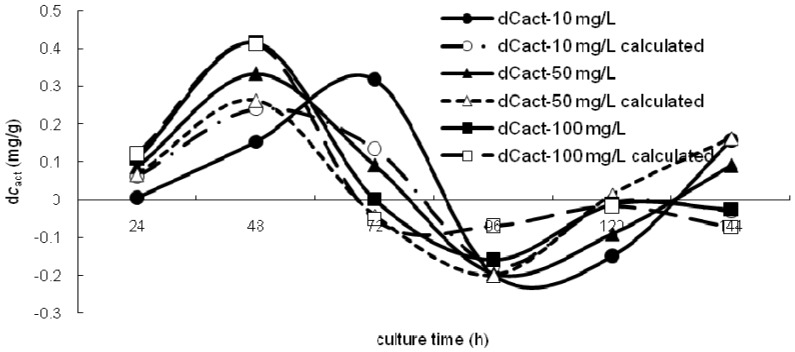
The d*c*_act_ and d*c*_act_ with calculated data for the bioaccumulation model.

In this model, three parameters *k*_pas_, *k*_act_ and *k*_Me_ were used to describe the effects of the three concentrations, *c*_pas_, *c*_act_ and *c*_Me_, on the bioaccumulation of the *S. spinosus* cells. An additional *c*_Me_ factor, compared to Chojnacka’s model, was added to describe the effects of the free strontium ion concentration in the solution on the bioaccumulation of the *S. spinosus* cells. Then, a dynamic programming approach was used to elaborate the experimental data. Figure 5 show that the modeled data could fit the experimental data well. The parameters that were used are listed in Table 4.

**Table 4:** The bioaccumulation model parameters.

Parameter		*k*_pas_	*k*_act_	*k*_Me_	*r*^2^
*c*_0_(Sr)	10 mg∙L^−1^	5.5784	−3.7626	0.0616	0.4936
	30 mg∙L^−1^	3.7022	−7.1359	0.0243	0.7292
	50 mg∙L^−1^	2.8420	−7.2902	0.0135	0.7628
	80 mg∙L^−1^	1.7891	−5.9332	0.0109	0.9570
	100 mg∙L^−1^	1.4800	−6.1437	0.0122	0.9316

The results showed that the concentration of strontium ions on the cell walls (*c*_pas_) was positively related to the strontium ion transport into the cytoplasm. Conversely, the concentration of strontium ions in the cytoplasm (*c*_act_) was negatively related to the strontium ions’ entrance into the cytoplasm. The reason for these relations may be that strontium ions on the cell walls cause a concentration stress that increases transport, but a high concentration of strontium ions in the cytoplasm may harm the cell, causing the cell to attempt to transport the ions out of the cytoplasm. Free strontium ions in the solution may exert a certain effect on bioaccumulation. Thus, this model can be summarized in the following manner:

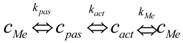
(19)


In this model, the free strontium ions may directly enter or be transported out of the cytoplasm into the culture medium by cell transmembrane transport processes [3,35]. This is different from the mathematical kinetics model proposed by Chojnacka and Wojciechowski [3] (Equation (17)). Their model shows that the free ions affected the bioaccumulation through the *c*_pas_, meaning that metal ions first bonded to cell wall and were then transported into the cytoplasm. There are many means of cell transmembrane transport. For example, P_1B_-type ATPases can transport a number of heavy metals ions, such as Cu^+^, Cu^2+^, Ag^+^, Zn^2+^, Cd^2+^, Pb^2+^ and Co^2+^, across biological membranes; these transporters are found in archaea, bacteria and eukaryote cells required for maintaining metal ions homeostasis [49,50]. However, these studies did not indicate the location of efflux ions on the cell wall or into the surrounding environment. Thus, future research needs to accurately measure the relationship between *c*_pas_, *c*_act_, *c*_Me_ and the cell transmembrane transport system activities.

## 4. Conclusions

*Scenedesmus spinosus* showed a strong capability for selective adsorption and bioaccumulation of strontium ions from simulated wastewater under culture conditions. The results showed that *S. spinosus* had a highly selective biosorption capacity for strontium with a maximum bioremoval ratio of 76%. The grey relational grade analysis showed that the order for different co-existing metal ion effects on strontium ion biosorption was Fe^3+^ > Cr^3+^ > Ni^2+^ > K^+^ > Cs^+^. The adsorbed strontium ion on cell walls was approximately 90% of the total adsorbed amount; the bioaccumulation in the cytoplasm varied by approximately 10%. The isotherm model Slips best fitted the experimental data and indicated that the adsorption of strontium ions onto *S. spinosus* cells was a complex process. The pseudo-second-order kinetic model suggested that adsorption was the rate-limiting step of the biosorption process. A new bioaccumulation model with three parameters *q*_pas_, *q*_act_ and *c*_Me_ was proposed and can yield a good fit with the experiment data. These results suggest that *S. spinosus* may be a potential biosorbent for treatment of nuclear wastewater under culture conditions.
